# Training gaps in trans and nonbinary health: Perspectives of resident physicians in Argentina

**DOI:** 10.1371/journal.pgph.0005863

**Published:** 2026-02-18

**Authors:** Carolina Silva, Paula Nalda, Valeria Mulli, Alfredo Eymann

**Affiliations:** 1 Division of Endocrinology, Department of Pediatrics, University of British Columbia, Vancouver, Canada; 2 Division of Clinical Pediatrics, Department of Pediatrics, Hospital Italiano de Buenos Aires, Buenos Aires, Argentina; 3 Instituto Universitario Hospital Italiano de Buenos Aires, Buenos Aires, Argentina; New York University, United States of America

## Abstract

Introduction and aims: A growing number of individuals identify as transgender and nonbinary (TNB), yet most healthcare professionals are not adequately prepared to address their health needs. Providers’ training is crucial to ensuring accessible, evidence-based care, and medical school and residency programs are a critical period to develop such knowledge and skills. Thus, we aimed to explore resident physicians’ perspectives on their training and competence caring for TNB individuals at a university hospital in Argentina. We conducted a cross-sectional study, between May and September 2024, among clinical and surgical residents. An anonymous questionnaire collected quantitative and qualitative data on training experiences, perceived competence, and comfort levels in caring for TNB patients. Descriptive statistics and thematic analysis were applied. A total of 173 residents participated; 89% completed undergraduate studies in Argentina, and 60% attended public universities. Only 24% felt adequately trained during medical school. Although 69% had treated TNB patients during residency, 54% thought their training was insufficient. Despite 84% feeling comfortable providing care, only 48% perceived themselves as competent. Notably, 84% agreed with the importance of education in TNB health, highlighting the need for comprehensive training, improved quality of care, and ensuring equity. These findings indicate that TNB health education remains insufficient across medical training programs in Argentina. There is a critical discrepancy between residents’ comfort and perceived competence in caring for TNB people. Participants recognized this gap and expressed strong support for enhanced curricular inclusion. Strengthening these educational foundations is key to transforming motivation into meaningful capability to advance equitable, affirming care across Argentina and Latin America.

## Introduction

Transgender identities encompass a diverse spectrum of experiences. The term “transgender” broadly refers to individuals whose gender identity does not align with the sex assigned to them at birth, challenge conventional gender roles, transition between genders, or identify outside the binary gender framework [1,2]. In this study, we use the term transgender and nonbinary (TNB) to inclusively refer to individuals with a wide range of gender identities—such as transgender, nonbinary, agender, gender fluid individuals, travesties, and others. We do so with the intention of recognizing and respecting the uniqueness of each identity and expression, while acknowledging the limitations and evolving nature of gender terminology. The estimated global proportion of TNB identities has traditionally been reported between 0.5% and 3% [3–5]. However, recent studies have reported a marked increase in the last decade, showing that up to 9% of adolescents have diverse gender identities [6,7].

TNB individuals have the right to receive healthcare that is respectful, comprehensive, and responsive to their specific needs. For more than a decade now, Argentina’s legislation has guaranteed the right to be identified according to each person’s gender identity, as well as access to comprehensive health services [8]. In line with this, national health guidelines for the care of TNB people are available, offering general recommendations to support access to healthcare [9]. Despite ongoing political debate and regulatory limitations —including recent restrictions on the provision of hormonal and surgical care to minors— education for healthcare providers and access to both general and gender-affirming healthcare TNB individuals remain essential and non-negotiable components of equitable medical practice [10–13].

Caring for TNB individuals has relevance across multiple clinical specialties. In pediatrics and adolescent medicine, healthcare providers play a key role in supporting youth as they navigate gender identity development, pubertal progression, and associated challenges or concerns. Endocrinologists, internal medicine specialists, and family physicians are often involved in providing gender-affirming medical care across the lifespan. Psychiatry and psychology contribute to promoting mental health, coping, and resilience. Gynecology and urology address reproductive and sexual health needs that may be distinct in TNB populations. Surgical teams provide essential interventions to align physical characteristics with gender identity. Nurses, social workers, speech and occupational therapists, among others, play vital roles in delivering inclusive, multidisciplinary care. As emphasized in international standards of care, gender diversity is not confined to specialized clinics but represents a fundamental aspect of comprehensive, person-centered healthcare across disciplines [11–13].

This breadth of clinical involvement, together with the proportion of TNB identities in the population, means that most healthcare professionals will encounter responsibilities related to the care of TNB individuals—whether for health issues connected to their gender identity or unrelated—throughout their practice. Medical school and residency programs represent a critical period for developing the knowledge, skills, and attitudes required to provide competent, inclusive care across diverse patient populations.

However, studies from various countries have demonstrated that many providers do not feel adequately prepared to care for TNB individuals [14–23]. The majority of this research has been conducted in North America, with a smaller but growing body of work emerging from Latin America. To-date, we have found no comparable studies in Argentina. Understanding the local context is essential to identifying specific needs and opportunities for improvement. Therefore, this study aimed to explore the attitudes and perceptions of resident physicians at a university hospital in Argentina regarding their training and perceived competence in providing care for TNB individuals.

## Methods

### Study setting

The study was conducted at a tertiary university hospital in Buenos Aires, Argentina, which offers over 50 residency programs across medical and surgical specialties and serves an urban, socioeconomically diverse population. Cultural attitudes toward gender and sexuality are heterogeneous, but overall, Argentina is regarded as one of the more socially progressive countries in Latin America. This country’s legal framework includes a Gender Identity Law [8]. Same-sex marriage has been legal since 2010. TNB health is formally recognized in national policy, with guidelines outlining standards of care and coverage for gender-affirming interventions within the public health system [9]. Informal provision of care is likely not a prominent issue, yet access barriers and inequities in care continue to be a significant concern [21,22]. Further, specific curricular standards for TNB health education remain limited, and institutional implementation is variable.

### Participants and procedures

The study population included resident physicians from all training years enrolled in core medical and surgical specialties. Residents participating in post-basic (subspecialty) training programs were excluded. Study invitations were sent via email. Participation was entirely voluntary, and all data were collected anonymously, ensuring confidentiality.

### Instrument

An anonymous electronic questionnaire was created in Google Forms. The questionnaire was informed by existing literature and national guidelines on TNB healthcare, and developed by a group of experts in medical education and TNB health to ensure content validity. In addition to expert review, the survey underwent pilot testing with five resident physicians to assess item clarity, comprehensiveness, and response burden, contributing to evidence of face and content validity. Necessary adjustments were made based on their feedback. As the role of these reviewers was limited to providing feedback for instrument refinement, with no data collection, formal consent was not required. No reliability testing was conducted.

The questionnaire included items on experience caring for TNB individuals, training received, self-assessed competence and comfort in providing care. Response formats varied by question and included yes/no/not sure options and Likert scales (ranging from “strongly agree” to “strongly disagree”). Additionally, several open-ended questions invited participants to elaborate on their perspectives around barriers to TNB individuals accessing health care, factors impacting their perceived competence, importance of incorporating this topic in medical education, and other suggestions, asking them to explain their reasoning for their chosen answer to the previous multiple-choice question. Basic demographic data (age, gender), specialty, year of residency, and type of university where participants completed their medical degree were also collected (S1 Appendix).

### Data analysis

Continuous variables were reported as means with standard deviations. Categorical variables were summarized using absolute and relative frequencies. To explore factors associated with resident physicians’ self-perceived competence in providing care to TNB individuals, we analyzed responses to the item “*I perceive myself as competent to care for transgender individuals*”. For this analysis, Likert-scale responses were dichotomized as “competent” or “not competent.”, and chi-square tests were conducted to assess associations between perceived competence and the following independent variables: gender (male/female), residency year (grouped as first and second versus third year or higher), specialty type (clinical versus surgical), and type of medical school attended (public versus private).

Qualitative responses were analyzed using an inductive thematic analysis approach. Two authors independently and iteratively reviewed the verbatim responses to identify emerging patterns and concepts. This was followed by an open coding process, during which identified categories were compared and discussed until consensus was achieved. This coding was done manually. Final thematic categories were refined and grouped to ensure internal coherence and representativeness of the data.

The questionnaire was conducted in Spanish; participants’ answers have been translated into English for reporting. Translations were reviewed by a bilingual member of the research team to ensure fidelity to participants’ intended meaning and cultural nuances in their answers to open-ended questions. Original responses in Spanish are available upon request.

### Ethics considerations

Approval was obtained from the Ethics and Protocols Committee of the University Hospital Italiano prior to the start of the study (approval number: 0016–23). The study involved healthcare professionals as participants and was deemed to pose minimal risk. Given the study’s minimal risk and the anonymous nature of the survey, the Ethics and Protocols Committee approved a waiver of written consent. Instead, informed implied consent was obtained: participants were informed, through the study invitation and survey introduction, about the purpose of the study, voluntary participation, confidentiality, and their right to withdraw at any time. Submission of the completed survey was considered evidence of consent to participate. The research was completed in accordance with the Declaration of Helsinki as revised in 2013 and relevant institutional and national ethical guidelines.

## Results

A total of 449 resident physicians from 20 core training programs were invited to participate, of whom 173 completed the questionnaire (response rate of 39%). Table 1 shows their characteristics. Most identified as female, and none chose “other” gender identities. The majority completed their undergraduate studies at national institutions (89%), most commonly public universities (60%). Most frequent specialties were pediatrics, internal medicine, and diagnostic imaging. There was comparable representation from each year of residency (Table 1).

**Table 1:** Resident Physicians’ Characteristics (n = 173).

Age, mean (SD)	28.7 (2.5)
**Gender**, n (%)	
Female	121 (70)
Male	53 (30)
Other	0
**Country of undergraduate medical degree**, n (%)	
Argentina	154 (89)
Other	19 (11)
**Type of undergraduate institution**, n (%)	
Public	103 (60)
Private	70 (40)
**Postgraduate Year,** n (%)	
1	40 (23)
2	42 (24)
3	38 (22)
4	34 (20)
5 (Chief resident)	19 (11)
**Specialty**, n (%)	
Pediatrics	38 (22)
Internal Medicine	23 (13)
Diagnostic Imaging	22 (13)
Obstetrics and Gynecology	19 (11)
Dermatology	16 (9)
General Surgery	12 (7)
Cardiology	8 (5)
Family Medicine	7 (4)
Gynecology	4 (2)
Ophthalmology	4 (2)
Cardiovascular Surgery	3 (2)
Intensive Care	3 (2)
Urology	3 (2)
Pediatric Surgery	2 (1)
Neurology	2 (1)
Orthopedics and Traumatology	2 (1)
Gastroenterology	1 (1)
Obstetrics	1 (1)
Anesthesiology	1 (1)
Otolaryngology	1 (1)
Radiation Oncology	1 (1)

SD = Standard deviation.

**Fig 1:**
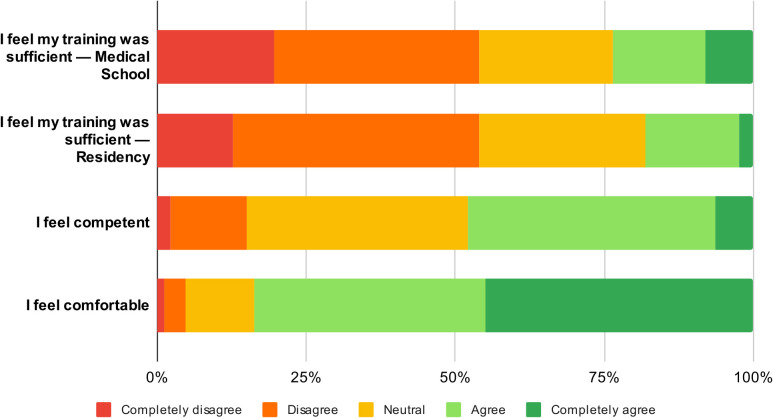
Resident physicians’ self-reported training adequacy, competence, and comfort levels in caring for TNB individuals (n = 173).

The majority of participants (93%) were aware of national laws or guidelines related to gender identity and care of TNB individuals. About half (51%) received some training on TNB health care during undergraduate education, but only 24% considered this training sufficient (Fig 1). During residency, 69% cared for TNB patients, mostly limited to less than five individuals (90%), and 53% participated in at least one academic activity related to TNB care. However, only 26% reported that their residency curriculum specifically included this topic, and 54% viewed their residency training as insufficient (Fig 1).

A strong majority (84%) supported the inclusion of TNB care in residency programs; 13% was neutral and only 4% was opposed to it. Participants emphasized the need for comprehensive training and the provision of equitable, high-quality, respectful care. It was noted: “It’s a necessary requirement as part of our medical training”; “We must treat all people the same way, regardless of their gender identity”; “It’s important to address consultations with sufficient tools and thus provide quality care”. Further, some referred to specific clinical needs of this population, and being capable to respond to these (Table 2).

**Table 2:** Residents’ perspectives: importance of training for the care of TNB people (n = 112).

Category	Quotes
**Providing high-quality care**	“Primary care can offer support with significant positive impact, especially if coordinated with public policies.” “Mainly to be able to provide accurate information during consultations and be equipped to address patients’ questions — and thus also ensure quality care.” “To provide low-barrier care and improve quality of life.” “All healthcare workers should receive training in this area as it cuts across all aspects of health. This doesn’t mean a cardiologist needs to know how to prescribe hormones, but they should know how to treat patients and how hormonal treatment might impact clinical care.”
**Comprehensive training for healthcare professionals**	“Otherwise, the training is incomplete, and we have to make up for it with extra courses/conferences outside of residency.” “Caring for trans people is something we will almost inevitably encounter, and I believe we should be well-prepared to do it with respect and appropriately.” “[…] one should be prepared to make health recommendations for all people.” “It is a necessary requirement as part of basic care.”
**Inclusive, compassionate approach**	“I believe we are seeing more and more trans patients, and it’s important to learn how to treat them so they feel comfortable and safe accessing healthcare.” “I think it’s important to have knowledge in order to best support all people. It’s important to ensure compassionate care, respect people’s identities, and support their health in all its aspects.” “Because all patients have the right to be treated with dignity during medical consultations, to be addressed and recognized as they wish.” “Everyone should be treated the same, regardless of gender identity.”
**Specific health issues of this population**	“They are a population with different needs, and we must be prepared to care for them.” “To know how to address and manage consultations with conditions specific to them.” “Their care tends to be more complex for this reason and due to the presence of conditions, for example, side effects of hormonal treatment.” “It is important to consider anatomical changes from hormone therapy and surgical gender-affirming procedures.”

Only 48% of participants felt competent to care for TNB individuals. Interestingly, 84% reported feeling comfortable doing so (Fig 1). The main barriers to competence were lack of specific training and limited practical experience. One participant described ”I have theoretical knowledge but I feel I lack practical training”, and another mentioned “We talk about trans patients but we are not given tools on how to treat them”. Facilitators included having received training, but also, their own personal attitude (interest in the topic, respectful approach and empathy). Themes and quotes are presented in Table 3.

**Table 3:** Residents’ perspectives: factors influencing their competence in caring TNB people (n = 120).

	Category	Verbatim
**Barriers**	**Deficient training**	“We talk about trans patients but are not given tools on how to treat them.” “I have little information about treatments, usual practices, and the legal framework for this group of patients.” “I would need more training to do it — I’d love to have that level of expertise.” “I only know generalities about how to address these patients, and I don’t think that’s enough to conduct a consultation/ assessment”
	**Lack of experience**	“I didn’t have the opportunity to care for or even be involved in caring for trans people” “I have theoretical knowledge but feel I lack practical training.”
**Facilitators**	**Sufficient training**	“I had sufficient education in medical school.” “I’ve acquired some tools during residency — for example, I know I should ask how they want to be addressed or that I should first call them by their last name.” “Even though it wasn’t extensive, I was able to learn a lot during my training and I feel I could provide adequate care for the patient.”
	**Empathy, open-mindedness**	“I understand the issues; there’s empathy.” “Patients should be treated equally, and their identity doesn’t change how I proceed.” “I treated them with respect, apologized if they felt uncomfortable, and listened to them.” “Holistic care means being competent from a human perspective.”
	**Personal interest**	“I trained outside the hospital in order to provide quality care to all patients, regardless of their gender identity.” “I’m interested in the topic and read about it.” “I always try to learn to be able to care for all kinds of patients.”

No statistically significant associations were found between perceived competence and sex (p = 0.5), residency year (p = 0.1), or specialty type (p = 0.09). However, a significant association was observed with the type of medical school: resident physicians who had graduated from private universities were more likely to perceive themselves as competent compared to those from public institutions (p = 0.009). The size of this effect was small to moderate (Cramer’s V = 0.1984).

Finally, when asked about access to healthcare for TNB people, 23% of participants were unsure, and 38% identified obstacles. Among these, deficient staff training, bias, institutional factors and negative experiences were the most commonly reported challenges. Table 4 shows main themes and participants’ quotes on this topic.

**Table 4:** Residents’ perspectives: Barriers to Health Care Access for TNB people (n = 65).

Category	Verbatim
**Lack of staff training**	“Ignorance about the reality of trans people and the positive impact health care could have on this population.” “Lack of knowledge about medical management and administrative/ legal considerations.” “Older doctors (a cultural barrier).” “Lack of knowledge among health workers about gender issues (gender vs. biological sex, asking about pronouns, providing information about their rights, etc.).”
**Bias**	“Rejection by professionals of new perspectives on gender, and their biases regarding hormonal and surgical treatment.” “Misinformation — stigmatization.” “It’s a taboo topic.”
**Institutional factors**	“The electronic medical record doesn’t represent gender identity visually like it does biological sex.” “The patient-calling system might make someone uncomfortable by announcing their full name.” “Historically, medicine has not been inclusive, and I don’t think there’s any active effort to include non-binary people. Even hospital posters or images don’t reflect them.”
**Negative experiences**	“Fear among trans patients of being mistreated by healthcare professionals.” “How we address the patient during visits.”

## Discussion

Equipping healthcare providers with the knowledge and skills to care for diverse patient populations is key to achieving equity and true inclusiveness in the healthcare system. To better understand the current needs and opportunities in our setting, we examined the experiences of resident physicians in Argentina, revealing significant educational gaps related to the care of TNB individuals. Concurrently, participants expressed overall comfort in working with this population, and support for targeted curricular integration of this topic.

These gaps in provider education do not exist in isolation—they reflect a broader landscape of structural and interpersonal inequities. Despite legal advances and growing societal recognition, TNB individuals continue to face obstacles to receiving respectful and competent health care [24–36]. Participants in our study echoed these concerns. Many pointed to deficient training of clerical staff and healthcare professionals, as well as institutional factors, such as inadequate electronic health record systems or rigid administrative processes. They also cited biases or past negative experiences as barriers deterring TNB individuals from seeking care. These findings are consistent with previous studies, which describe TNB individuals avoiding healthcare settings out of fear of being disrespected, misunderstood, or exposed to discrimination [33–36].

In this context, healthcare providers play a central role not only in delivering care but also in creating affirming and safe environments [37,38]. Our findings mirror a well-documented global trend: the near-absence of TNB-related content in formal medical curricula [39–42]. Most participants reported little to no structured training during medical school or residency, leaving them to rely on personal values rather than appropriate teaching. With this, it was not surprising that relatively few felt competent to care for TNB patients. Many expressed the need for practical tools, structured guidance, and more opportunities for clinical exposure, which underscores the importance of not only increasing TNB content in curricula, but also designing it in a way that supports applied skill development.

Comparable findings have been described in other Latin American contexts, where efforts to integrate gender and sexual diversity into medical education remain limited and uneven. Studies from Chile, Brazil, and Mexico have reported similar gaps in curricular content, lack of faculty training, and discomfort among health professionals when addressing gender and sexual minorities [21–23]. Taken together, these patterns point to the need for developing locally relevant training approaches that consider sociocultural and institutional realities.

Resident physicians’ insights in this study suggest that current training models are influenced by longstanding cultural and institutional norms within medical education. Participants’ emphasis on learning these topics independently, and being inclined to provide this care just based on their own personal values, likely reflects a broader system where formal instruction on gender and sexuality is limited, and where implicit expectations remain largely cis- and heteronormative. These perspectives align with previous analyses describing Latin American medical education as historically shaped by patriarchal structures, binary understandings of sex and gender, and a focus on biomedical rather than sociocultural aspects of care [24,25]. In such contexts, inclusivity often depends on individual initiative rather than institutional commitment. Recognizing and addressing this hidden curriculum is essential to ensure that educational reform goes beyond content addition to encompass cultural and systems change within training environments [38–42].

Interestingly, we did find a statistically significant association with the type of medical school attended: resident physicians who had graduated from private universities were more likely to perceive themselves as competent. Although the effect size for this finding is small to moderate, it remains meaningful, particularly in social and educational research, where effects are often modest yet carry important implications for practice and policy. This finding warrants further exploration. It is possible that private institutions may offer more comprehensive or explicit training on gender-affirming care, or that students at these institutions have access to different clinical exposures, resources, or support systems that contribute to greater confidence in this area. Alternatively, perceived competence may reflect differences in institutional culture, or levels of faculty engagement with issues of gender diversity.

A notable finding from our study is the mismatch between resident physicians’ comfort and their perceived competence. Despite the challenges previously described, the vast majority of our study participants felt comfortable providing care to TNB people. This discrepancy—comfort without competence—represents a critical distinction. Notably, the degree of comfort reported by resident physicians in our study appears higher than in comparable international research [17,43,44]. Contextual factors unique to Argentina, such as the country’s long-standing legal recognition of gender diversity, progressive social attitudes, or even generational shifts in values among trainees, may help explain this finding. Together, these elements may foster a more affirming baseline for engagement with TNB patients, even in the absence of comprehensive training.

There was also strong support among participants for formally incorporating TNB health into residency programs. Resident physicians emphasized multiple reasons for this: to meet their own learning needs, to address the specific healthcare concerns of TNB individuals, and to fulfill their ethical responsibility to provide inclusive, holistic, high-quality care. These views are encouraging and suggest a readiness among early-career physicians to engage with gender diversity content in meaningful ways. Similar calls for curriculum reform have emerged in other countries, where learners are increasingly advocating for more inclusive, socially responsive medical education [41,42,45,46]. Personal motivation was identified by our study participants as an important factor in achieving competence, particularly, qualities such as empathy, open-mindedness, and personal interest; but at the same time, they acknowleded that these alone are insufficient for delivering clinically sound care.

## Limitations

This study has some limitations. First, it was conducted at a single academic institution, and while it includes a broad sample of specialties and resident physicians from different training backgrounds, findings may not be fully generalizable. Second, the study was conducted prior to recent policy changes affecting gender-affirming care in minors, which may influence both training opportunities and attitudes moving forward, particularly considering the strong representation of pediatric residents in our sample [10]. Third, as with all survey-based studies, there is potential for self-selection bias, partly mitigated by response rates that are higher than similar survey-based studies [40]. Participants who chose to complete the survey may have been more interested in TNB health topics than non-respondents, potentially leading to overestimation of comfort or competence levels. Social desirability bias may also have affected self-reported attitudes, especially given the sensitivity of the topic. Finally, while the questionnaire underwent expert review and pilot testing for face and content validity, additional psychometric evaluation, such as construct or criterion validity testing, was beyond the scope of this exploratory study. Future research could strengthen these aspects through formal validation in larger and more diverse samples.

## Implications and future research

These findings point to clear directions for educational and institutional reform. Competency-based objectives that integrate conceptual understanding with supervised clinical experience in TNB care can help transform awareness into applied skill. Faculty development and institutional policy initiatives are equally important to build capacity and sustain inclusive practice. The comfort–competence gap identified here underscores that motivation and empathy, while necessary, are not sufficient for readiness to practice. Bridging this gap will require coordinated efforts in curriculum design, faculty training, and institutional commitment, providing a foundation for broader reform across Latin America.

Further studies should explore the perspectives of faculty, curriculum leaders, and TNB patients themselves. Qualitative research involving in-depth interviews or focus groups could complement these findings and guide the development of context-specific educational interventions. Ultimately, aligning medical education with the needs and rights of TNB individuals is essential to ensuring truly equitable care.

## Conclusion

This study reveals a substantial disconnect between resident physicians’ comfort and their perceived competence in caring for TNB individuals, calling for more comprehensive and practical training. The strong support among participants for including TNB health in medical education presents a timely opportunity to strengthen curricula and better prepare future healthcare professionals to provide respectful, informed, and affirming care. These findings offer an actionable foundation for curriculum development and faculty training in Argentina, and may inform similar initiatives in other Latin American contexts where comparable gaps persist.

## Supporting information

S1 Appendix Survey questions and response options. (DOCX)
