# Supplementary material for: Training gaps in trans and nonbinary health: Perspectives of resident physicians in Argentina
Source: PLOS Glob Public Health. 2026 Feb 18;6(2):e0005863. doi: 10.1371/journal.pgph.0005863 (PMC12915949; doi:10.1371/journal.pgph.0005863)
Supplement: S1 Appendix — (DOCX) [file pgph.0005863.s001.docx]

S1 Appendix. **Survey questions and response options**

| **Age (years)** | Free text | |
| --- | --- | --- |
| **Gender** | Multiple choice | 1. Male 2. Female 3. Other 4. Prefer not to answer |
| **Specialty** | Free text | |
| **Year of residency** | Free text | |
| **Type of institution where you completed your medical degree** | Multiple choice | 1. Public 2. Private |
| **Did you complete your medical degree in a country different to Argentina?** | Dichotomous response | 1. Yes 2. No |
| **During medical school, did you receive training in the care of trans and non-binary individuals?** | Dichotomous response | 1. Yes 2. No |
| **During your current residency program, have you participated in the medical care of trans or non-binary individuals?** | Dichotomous response | 1. Yes 2. No |
| - **If your answer was yes, approximately how many trans or non-binary patients have you cared for during your residency?** | Free text | |
| **During your current residency program, have you participated in any activity (class, case conference, multidisciplinary meeting) in which the care of trans and non-binary individuals was discussed?** | Dichotomous response | 1. Yes 2. No |
| - **If your answer was yes, how many times have you participated in such activities? Please provide an approximate total number of hours** | Free text | |
| **Do you know if the care of trans and non-binary individuals is included in your training program's curriculum?** | Multiple choice | 1. Yes 2. No 3. Not sure |
| **Are you familiar with the legal framework regarding gender identity and the care of trans and non-binary individuals?** | Multiple choice | 1. Yes 2. No 3. Not sure |
| **Do you believe there are institutional barriers that limit access to healthcare for trans and non-binary individuals?** | Multiple choice | 1. Yes 2. No 3. Not sure |
| - **If your answer was yes, in your opinion, what are the main barriers?** | Free text | |
| **Do you believe you will care for trans and non-binary individuals during your professional practice after completing your residency?** | Multiple choice | 1. Yes 2. No 3. Not sure |
| **Do you believe training in the care of trans and non-binary individuals should be included in your residency program** | Multiple choice | 1. Strongly agree 2. Agree 3. Neither agree, nor disagree 4. Disagree 5. Strongly disagree |
| - **If you wish, explain your reasoning** | Free text | |
| **Do you consider the training you received in medical school regarding the care of trans and non-binary individuals to be sufficient?** | Multiple choice | 1. Strongly agree 2. Agree 3. Neither agree, nor disagree 4. Disagree 5. Strongly disagree |
| **Do you perceive yourself as competent to care for the health of trans or non-binary individuals?** | Multiple choice | 1. Strongly agree 2. Agree 3. Neither agree, nor disagree 4. Disagree 5. Strongly disagree |
| - **If you wish, explain your reasoning** | Free text | |
| **Do you feel comfortable caring for trans or non-binary individuals?** | Multiple choice | 1. Strongly agree 2. Agree 3. Neither agree, nor disagree 4. Disagree 5. Strongly disagree |
| - **If you wish, explain your reasoning** | Free text | |
